# COILUMICA: coil embolization of a coronary artery to pulmonary artery fistula via novel dual lumen micro catheter technique

**DOI:** 10.1186/s42155-020-00177-4

**Published:** 2020-11-19

**Authors:** Mithun Nambiar, Julian Maingard, Kenny Li, Lee-Anne Slater, Ronil V. Chandra, Winston Chong, Duncan Mark Brooks, David McGaw, Hamed Asadi

**Affiliations:** 1 Interventional Neuroradiology Unit, Monash Imaging, Monash Medical Centre, 246 Clayton Road, Clayton, VIC 3168 Australia; 2 Faculty of Medicine, Nursing and Health Sciences, Monash University, Wellington Road, Clayton, VIC 3800 Australia; 3 School of Medicine, Faculty of Health, Deakin University, 75 Pigdons Road, Geelong, VIC 3216 Australia; 4 Interventional Neuroradiology Unit, Department of Radiology, Austin Hospital, 145 Studley Road, Heidelberg, VIC 3084 Australia; 5 Interventional Service, Monash Heart, Monash Medical Centre, 246 Clayton Road, VIC 3168 Clayton, Australia

**Keywords:** Coronary vessel anomaly, Fistula, Embolization, Endovascular, Percutaneous cardiac intervention, Radiology, Microcatheter

## Abstract

**Background:**

Management of coronary artery fistula (CAF) is based on obliterating the fistula communication between the cardiac arteries and other thoracic vessels.

**Case presentation:**

We describe the presentation of an 85-year-old female with progressive exertional dyspnea on a background of a long standing left anterior descending diagonal to pulmonary artery fistula. We utilized neuro-interventional techniques to perform coil embolization via use of a Scepter XC dual lumen micro catheter.

**Conclusions:**

Dual lumen balloon catheters allow for super-selective artery interrogation, stability of balloon positioning, with less trauma to vessel architecture and accurate embolization. There were no complications and the patient reported improvement of symptoms on review.

## Background

Coronary artery fistulae (CAF) were first described by Krause in 1865 (Krause 1865). They are rare occurrences that involve an abnormal connection between a coronary artery and cardiac chamber or adjacent major thoracic vessel (Yamanaka and Hobbs 1990). The majority of incidental CAF are small and clinically silent. Symptomatic patients may warrant surgical or interventional approach to ligate or embolise the fistula.

The treatment of dural arteriovenous fistulae and cerebral aneurysms with liquid embolic agents or coil embolization, are well established in the neuro-interventional literature (Heit et al. 2015; Jagadeesan et al. 2018a; Wallace et al. 2019). Distal access is often gained using microcatheter techniques. Balloon occlusion catheters allow for safe and efficacious occlusion of fistulous communications (Heit et al. 2015; Wallace et al. 2019). The use of neuro-interventional equipment and technique for the treatment of peripheral and cardiac fistulae is rare and only once previously reported (Jagadeesan et al. 2018b). This case outlines the application of neuro-interventional techniques to a difficult left anterior descending diagonal artery (LADD) to pulmonary artery (PA) fistula and outlines the benefits of a multidisciplinary approach to complex cases across cardiac and neuro-interventional teams.

## Case report

**Fig. 1:**
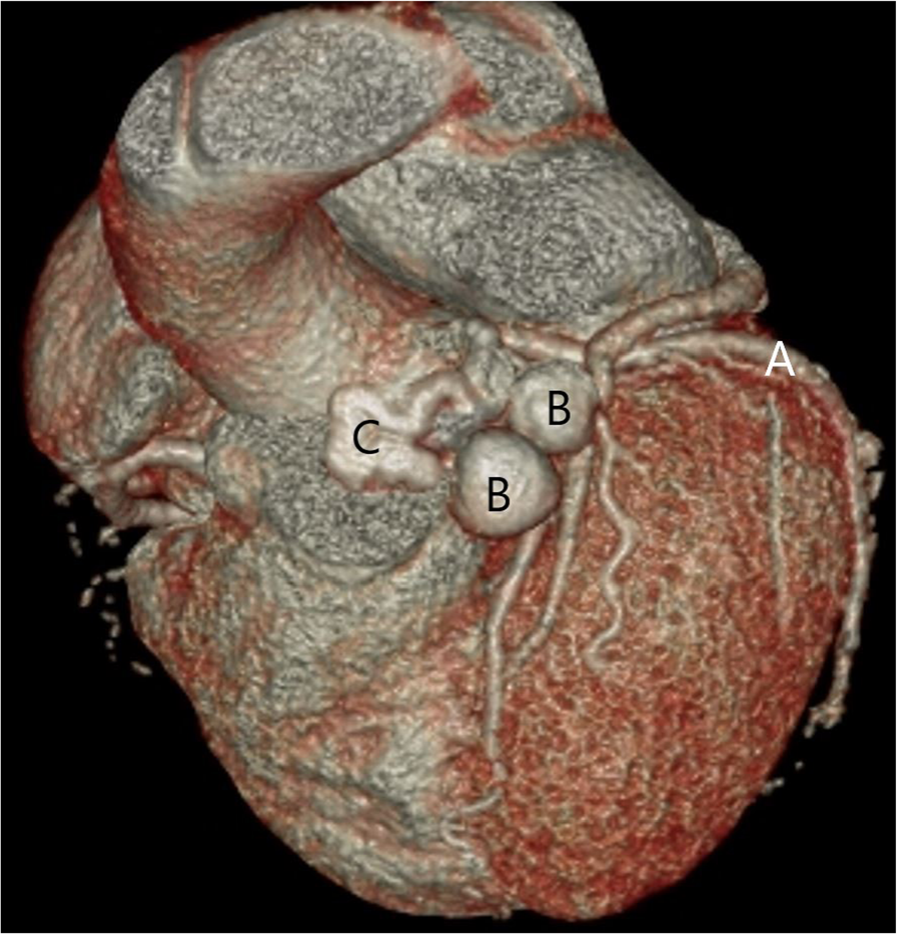
3D reconstruction from a coronary CTA demonstrating: **a** LADD, **b** flow related aneurysms, **c** tortuous artery fistula

**Fig. 2:**
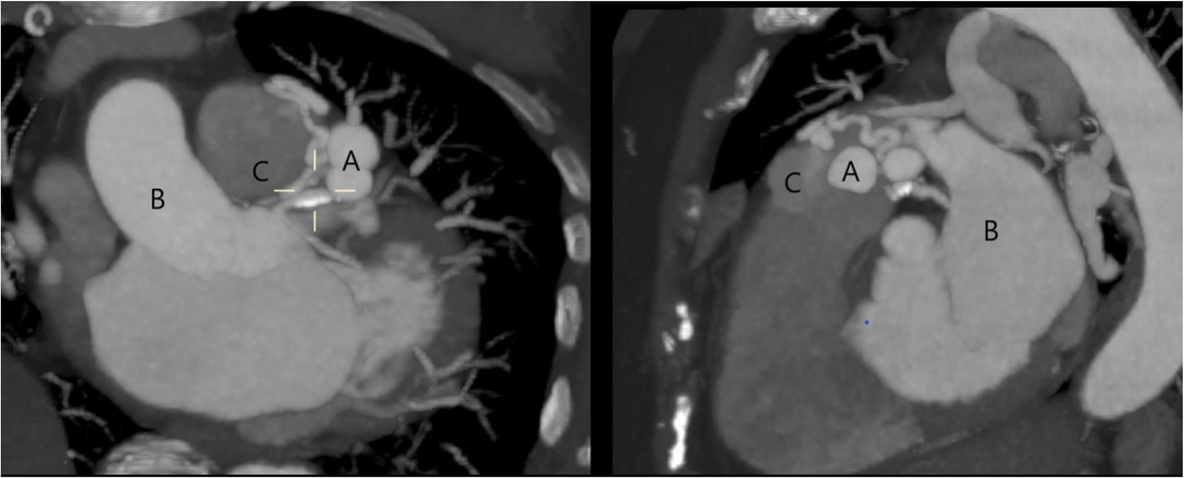
Multiplanar reformat (MPR) images of the coronary CTA demonstrating: **a** inflow aneurysms from LADD and artery fistula, **b** aorta, **c** pulmonary artery with a subtle contrast blush as a result of abnormal communication from LADD to PA

An 85-year-old female presented with symptoms of progressive exertion dyspnea, on a background of a known long-standing LADD to PA fistula (Figs. 1 and 2). Given concerns for a possible high flow shunt with associated steal phenomenon on a recent diagnostic catheter angiogram, she was referred for endovascular embolization. There were two large in-line flow related aneurysms measuring 14 mm and 12 mm respectively, with an additional supply to the second aneurysm from a small septal branch. There was high flow shunting into the pulmonary artery. A multidisciplinary approach was taken between Interventional Neuroradiology and Interventional Cardiology teams.

**Fig. 3:**
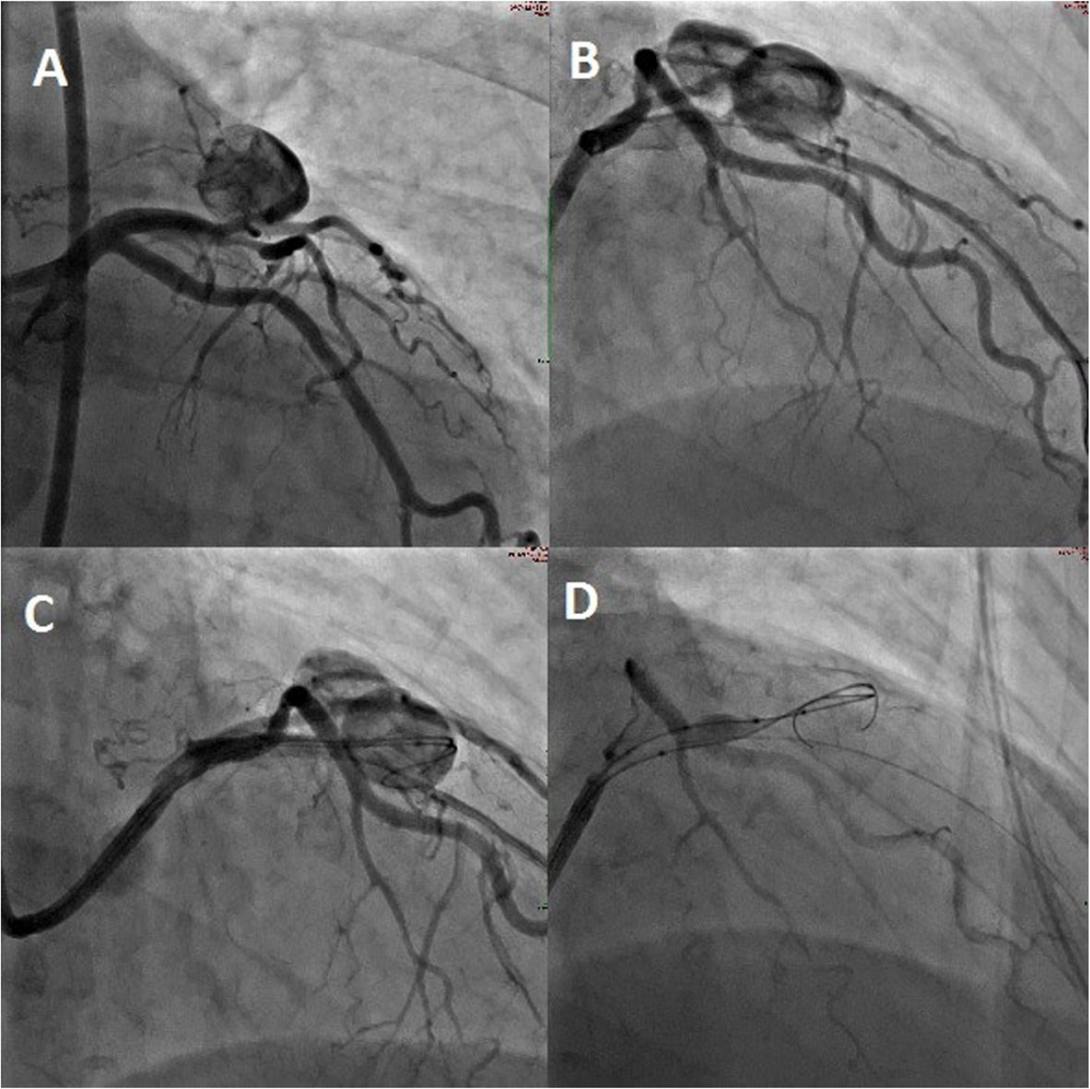
**a** and **b** Initial angiography performed via the 8French Judkins catheter revealed an LADD to pulmonary artery fistula with two large communicated flow related aneurysms. Moderate morphologically significant LADD stenosis was present prior to assessment with an FFR wire. **c** Interrogation with a Scepter XC dual lumen balloon catheter and Headway 21 microcatheter was performed **d** Inflation of the Scepter XC revealed dominant contribution from the LAD feeder without significant high flow supply from elsewhere

**Fig. 4:**
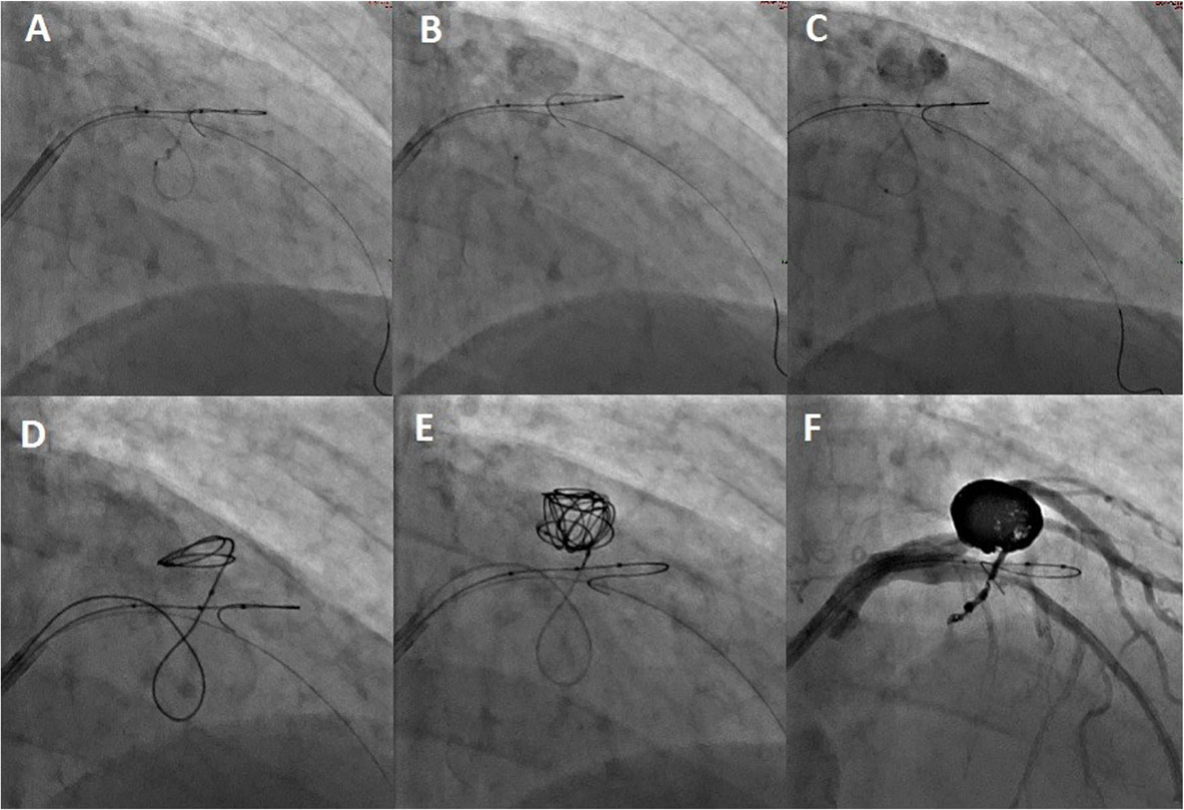
**a** Superselective angiography via the Headway 21 microcatheter reveals a small septal feeder with flow into the large flow related aneurysm. **b** This was better characterised with inflation of the Scepter XC in the parent LAD occluding the dominant arterial feeder and reducing competitive flow **c** the microcatheter was advanced into the aneurysm sac and **d** - **f** multiple neurovascular coils of varying sizes were deployed to occlude the sac and inflow septal branch

**Fig. 5:**
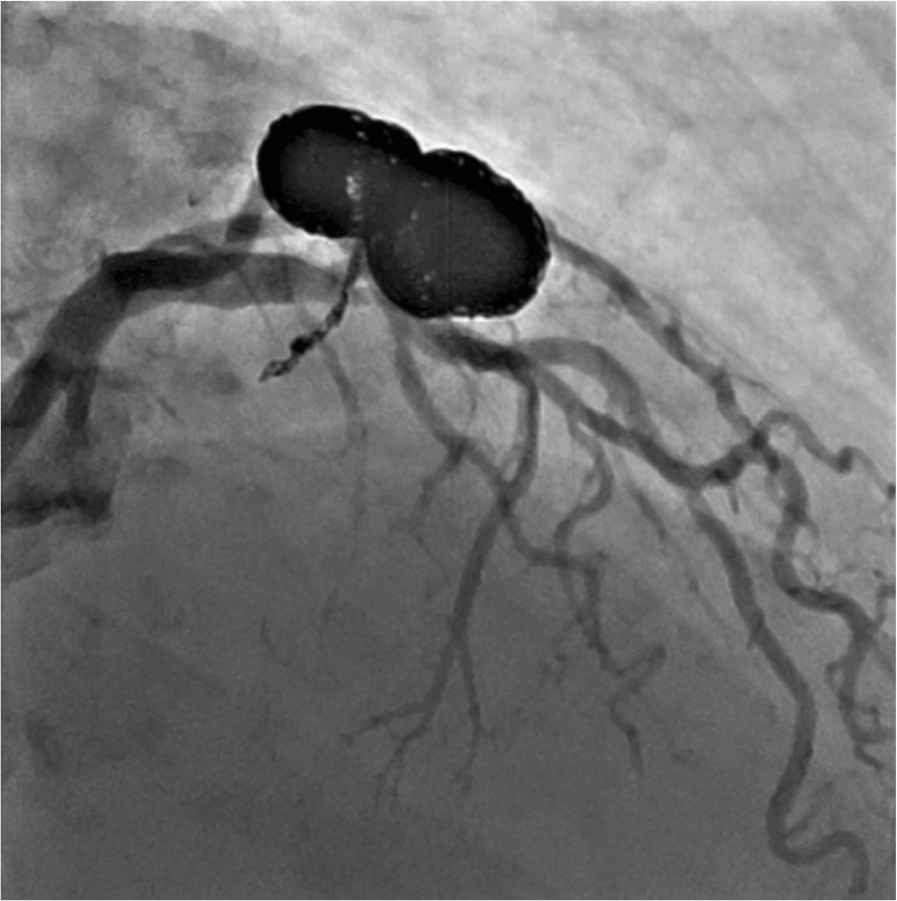
Post-interventional angiogram. The additional aneurysm sac was coiled via the dominant feeder arising from the LAD. Complete obliteration was achieved

Under conscious sedation and local anaesthetic an 8 French common femoral artery sheath (Terumo, Tokyo, Japan) was inserted. Diagnostic angiography was performed using an 8 French Judkins catheter (Cordis, CA, USA) (Fig. 3) with super-selective angiography and characterization of the fistula compartments (Fig. 4) using a dual lumen Scepter XC balloon microcatheter (Microvention, CA, USA) over a 0.014 in. Synchro standard microwire (Stryker Neurovascular, CA, USA). The fistula feeders and aneurysmal compartments were selectively interrogated with intermittent balloon inflation to further understand the morphology of the lesion and identify additional arterial supply. A dominant feeder was seen to supply a large flow related aneurysm which arose from the LAD. A second flow related aneurysm arose from a minor septal branch from the LAD. Following this, multiple Target 360 neurovascular coils (Stryker Neurovascular, CA, USA) were used to embolise the flow related aneurysms and occlude the fistula via a Headway 21 microcatheter (Microvention, CA, USA). Larger framing coils were used to occlude the ostium of the first aneurysm from the dominant arterial feeder from the LAD. The small septal supply was sacrificed in order to fully exclude the second aneurysm. Post-interventional angiography demonstrating adequate coil obliteration of the fistula is demonstrated in Figs. 4 and 5. Both pre and post intervention fractional flow reserve (FFR) measurements were unremarkable (0.89 pre-intervention versus 0.82 post-intervention). As such no additional intervention was performed. The procedure was well tolerated and there were no complications. The groin was closed with a 6 French Proglide (Abbott, IL, USA).

The patient was well at 1 month clinical review with improvement in symptoms and no further delayed complications.

## Discussion

The first percutaneous transluminal closure of a coronary artery fistula was described in 1989 (Nguyen et al. 1989). Mavroudis et al., described conditions for successful embolization as being able to safely cannulate the coronary artery branch supplying the fistula, to protect the mother vessel; to select patients with absence of multiple CAF connections; and the presence of single narrow drainage site into chamber/vessel (Mavroudis et al. 1997). Potential complications with transcatheter embolization include coil migration resulting in distal embolization, coronary spasm, cardiac arrhythmia, fistula dissection and thrombosis (Jama et al. 2011; Armsby et al. 2002).

Balloon assisted embolisation is a common neuro-interventional procedure, first described in 1997 (Moret et al. 1997). For cerebral aneurysms with a large neck, the balloon is inflated across the neck, which stabilizes the coiling microcatheter during coil embolization, and also protects branch vessels arising from the aneurysm neck (Wallace et al. 2019). If using an embolic agent, inflation of a balloon prior to injection, slows the flow into the feeding vessel and thereby reduces the risk of excessive anterograde penetration of the embolic agent (Liang et al. 2013).

Jagadeesan et al. (Jagadeesan et al. 2018b) has previously described the use of a Scepter C dual lumen balloon micro catheter (Microvention, CA, USA) to treat a RCA to PA fistula via a liquid embolic agent (Onyx, Medtronic, MN, USA). We describe the use of the Scepter XC micro catheter (Microvention, CA, USA), which is a dual lumen compliant balloon catheter that has been effectively used in neuro-intervention for cerebral aneurysms (Wallace et al. 2019). The second lumen of a dual lumen catheter accommodates an 0.014 in. diameter microwire which is torquable and steerable in interrogating tortuous vessels (Wallace et al. 2019).

The use of detachable neurovascular coils allows for controlled placement and assessment of flow in non-target vessels prior to deployment. The enhanced potential for navigation of the microwire allows for super-selective interrogation of small feeder vessels which may otherwise be missed due to competitive arterial flow. Therefore the Scepter XC is useful for super-selective interrogation that is useful in tortuous cardiac vessels as in our case.

The compliance of the Scepter XC balloon has previously been documented in the treatment of cerebral vasospasm, where radial expansion of the balloon conforms to the course of the vessel, which allows for balloon inflation that is less traumatic to the vascular architecture to prevent arterial injury and vessel rupture (Heit et al. 2015). The compliant nature of the balloon has also been noted to be useful for angioplasty at branch points in a vessel, where the balloon slightly prolapses into branching vessels, thereby conveying greater stability to the balloon (Heit et al. 2015). This feature is important for stable access in tortuous cardiac anatomy. As in our case, the ability to selectively coil flow related aneurysms while impeding forward flow, allows for more stable placement of the embolic coil.

Furthermore, the balloon and microwire lumens are separate in the Scepter XC micro catheter, which allows for retraction, exchange or reposition of the microwire without losing position of the catheter. This is not possible in a single lumen catheter due to subsequent retrograde blood flow into the balloon that can hamper inflation and deflation of the balloon (Wallace et al. 2019).

A potential shortfall of using the Scepter XC catheter, however, is that the 5 mm nose distal to the balloon can make positioning of the catheter difficult in areas of vessel bifurcation or tortuosity (Heit et al. 2015). Furthermore, the preparation of the Scepter XC balloon, to ensure the absence of air in the device, has been noted to be more time consuming than other balloon catheter devices (Heit et al. 2015). However, these issues did not affect our interventional procedure.

The patient was well at six-month clinical review with improvement in symptoms and no delayed complications.

## Conclusion

The management of CAF is based on obliterating the fistula communication between cardiac arteries and other vessels. Dual lumen balloon catheters allow for super-selective artery interrogation and more accurate embolization, while reducing flow into a shunting lesion. Our approach took advantage of the Scepter XC balloon’s compliance which provided greater stability in balloon positioning and can reduce trauma to vessel architecture.

## Data Availability

Not applicable.

## Abbreviations

CAF: Coronary artery fistulae
LADD: Left anterior descending diagonal artery
PA: Pulmonary artery
FFR: Fractional flow reserve
